# Alterations in CNS Functions and DNA Methylation in Rats after 24 h Exposure to Peat Smoke

**DOI:** 10.3390/toxics9120342

**Published:** 2021-12-08

**Authors:** Vera A. Vokina, Larisa M. Sosedova, Mikhail A. Novikov, Viktor S. Rukavishnikov, Ekaterina A. Kapustina, Anton N. Alekseenko, Elizaveta S. Andreeva

**Affiliations:** FSBSI East-Siberian Institute of Medical and Ecological Research, 665827 Angarsk, Russia; sosedlar@mail.ru (L.M.S.); novik-imt@mail.ru (M.A.N.); rvs_2010@mail.ru (V.S.R.); kapustinkae@yandex.ru (E.A.K.); alexeenko85@mail.ru (A.N.A.); liza.2995@mail.ru (E.S.A.)

**Keywords:** peat smoke, rats, EEG, behavior, DNA fragmentation and methylation

## Abstract

The use of a developed experimental model of a natural fire made it possible to assess the consequences of 24 h exposure to peat combustion products in albino rats. Peat smoke exposure leads to behavioral disturbances in rats, characterized by an increase in locomotor activity and an increased level of anxiety. Indicators of brain bioelectrical activity of the exposed animals supported the state of anxiety and psychoemotional stress. Epigenetic changes in the blood cells of exposed animals were revealed under 24 h exposure to peat smoke, characterized by a decrease in the level of global DNA methylation.

## 1. Introduction

Peat fires, reported in many parts of the world, with the largest in Indonesia [1,2], Russia [3], the USA [4,5,6,7], and Africa [8], are becoming a global problem. This type of natural fire is extremely difficult to extinguish, despite heavy rain, snow, cold weather, and human attempts to control it, and its duration can range from a week to several years [9]. During peat fires, combustion products with a high content of fine particles, polyaromatic hydrocarbons, and toxic compounds are released from the upper soil layers and enter the atmosphere. It is known that wildfire smoke spreads over long distances and crosses geographic boundaries, affecting areas far away from the original source [10]. Toxic peat smoke poses a significant risk to human health and is an urgent problem for densely populated regions [11,12]. A few investigations are devoted to the study of the toxic properties of smoke from peat fires. They mainly target the pulmonotoxic effects of acute exposure to particulate matter [10,11]. Although air pollution from wildfire smoke is known to cause respiratory irritation and damage, its effect on the central nervous system is not well described. The problem of the adverse mental health effects caused by particulate matter is of particular relevance [13,14,15]. Current evidence links wood smoke exposure to cognitive dysfunction, including dementia and Alzheimer’s disease [16]. Although the mechanisms underlying air pollution-induced CNS damage are poorly understood, new evidence suggests that microglial activation and changes in the blood–brain barrier are key elements in this process. Ku et al. suggested that miR-574-5p is a potential intervention target for the prevention and treatment of PM_2.5_-induced neurological disorders [17]. Recently, an increasing number of publications have appeared indicating the presence of epigenetic changes caused by toxicants of various chemical structures in low doses that do not lead to mutations [18]. In this regard, the problem of the long-term consequences of wildfire smoke exposure and accumulated chemical load from parents is gaining importance. Environmental epigenetics focuses on cell or organism responses to environmental factors, creating altered phenotypes or diseases. In this case, epigenetic modifications can be one of the mechanisms for the formation of long-term consequences of the effect of chemical stress induced by smoke from wildfires on rats and on the postnatal development of their offspring. It is of particular interest to study the neurotoxic effects of peat smoke and its effect on the level of DNA fragmentation and methylation in a model experiment.

## 2. Materials and Methods

### 2.1. Animals and Experimental Design

Experimental studies were carried out on 40 3-month-old outbred albino Wistar male rats (weight: 180–200 g). All the animals were kept under a 12/12 h light/dark cycle, on a ventilated shelf, and under controlled temperature and humidity conditions (22–25 °C and 55–60% humidity). Experimental animals were obtained from the vivarium of Federal State Budgetary Scientific Institution “East Siberian Institute of Medical and Ecological Research” (FSBSI ESIMER) and kept on a standard diet (BioPro Russia, water ad libitum). All animal experiments were approved by the ethical committee of FSBSI East-Siberian Institute of Medical and Ecological Research (identification code: E32/19; date of approval: 10 September 2019, amended/approvals every 6 months) and carried out in compliance with the rules of humane treatment of animals in accordance with the requirements of the International Recommendations for Biomedical Research Using Animals (WHO, Geneva, Switzerland, 1985), U.K. Animals (Scientific Procedures) Act (UK, 1986) and National Institutes of Health guide for the care and use of Laboratory animals (NIH Publications No. 8023, revised 1978).

Animals of experimental group (*n* = 20) were exposed to peat smoke inhalation for 24 h. The control group (*n* = 20) was supplied only clean air without smoke, but other environmental conditions in the chamber were the same for both control and test exposure groups. One day after the end of exposure, the animals were tested in an open field in the Morris water maze, and an EEG examination and assessment of the level of fragmentation and global DNA methylation in blood cells were carried out.

### 2.2. Exposure Study

Experimental modeling of the impact of peat smoke was carried out in exposure chambers with a volume of 200 L. The smoke emitted from the smoke generator was directed into exposure chamber equipped with air temperature and humidity control devices, in which the animals were kept [19]. A fan was used to maintain a uniform flow of smoke into the breathing area of the animals. Peat samples were taken in ecologically clean area at the site of a drained peat bog, far from an industrial zone. Peat sampling was carried out in accordance with GOST R 54332-2011 “Peat. Methods of sampling”. Sampling was carried out manually from the bottom of the holes dug to a depth of at least 0.4 m from the surface. The collected peat samples were stored in a room with standard temperature and humidity without direct access to sunlight. A general qualitative analysis of the air in the chamber was carried out using an Agilent 5975 gas chromatographic mass spectrometer (Agilent Technologies, Santa Clara, CA, USA). The general qualitative composition of the air was carried out by taking an air sample onto a microfiber by solid phase microextraction (SPME) for 10 min. Then, the microfiber SPME was analyzed on a gas chromatography–mass spectrometer. Separation of the components was carried out on an HP-5MS capillary column (30 m, 0.25 mm, 0.25 μm) in a temperature gradient mode. The scanning range of masses is 30–500 atomic mass unit (amu).

Qualitative analysis for volatile aldehydes was carried out using the reagent o-pentafluorobenzylhydroxylamine (PFBHA). For this, air samples were taken into distilled water. The selected aldehydes in distilled water were derivatized with the o-PFBHA reagent; the derivatized aldehydes were extracted with hexane. The hexane extract was analyzed using the gas chromatography–mass spectrometer. The mass scanning range is 35–500 amu. Components were identified using the NIST mass spectra library.

The mass concentration of PM_2.5_ was measured with a Kanomax 3521 piezo-balanced dust monitor (Kanomax, Andover, New Jersey, USA). Concentration of carbon monoxide (CO) was analyzed using a Chromos GC-1000 gas chromatograph (Chromos Engineering, Moscow, Russia). Concentrations of NO_2_, SO_2_, formaldehyde, furfural, and acetaldehyde were measured with a PE-5300VI spectrophotometer (Ecroskhim, St. Petersburg, Russia).

### 2.3. Open-Field Test

Testing was carried out in a round arena with a diameter of 40 cm, a translucent floor, and walls 25 cm high with inverted lighting using 3D animal tracking system “EthoStudio” (Novosibirsk, Russia) [20]. The horizontal locomotor activity (distance run) and time in the center were measured automatically. The number of rearing, grooming, freezing, and defecation episodes during a 3 min period was measured manually. The time in the center was recorded as a percentage of the total observation time.

### 2.4. Morris Water Maze

The used Morris water maze was a circular pool 1.5 m in diameter and 60 cm in height, filled with water at a temperature of about 25 °C to a height of 25 cm and clouded by the addition of chalk. The top surface of the hidden platform was 14 cm in diameter and 1.5 cm below the surface of the water. Animals were tested four times (with an interval of 60 s) sequentially from different sectors of the pool, while the location of the platform hidden under the water remained constant. If the animal did not find the platform within 60 s, it was forcibly placed on it. The residence time on the platform was 60 s. Mean escape latency (in seconds) to reach the hidden platform was recorded.

### 2.5. EEG Measurements

EEG recording was performed using a portable 8-channel electroencephalograph “Neuron-Spectrum-1/V” (Neurosoft, Ivanovo, Russia). We used thin needle electrodes: (1) two recording electrodes were introduced subcutaneously in the parietal part of the head, in the left and right sides of the brain; (2) reference electrode was introduced subcutaneously in the area of nasal bone; (3) ground electrode was fixed on the tail. The EEG was recorded in background test mode for 60 s. The boundaries of high- and low-frequency filters were 0.5 and 35 Hz; sampling frequency was 200 ^с–1^. Automatic artifact removal was used to exclude any epochs with remaining artifacts. Clean epochs were subjected to fast Fourier transform (FFT) to obtain absolute spectral power in the delta (0.5–4.0 Hz), theta (4.0–8.0 Hz), alpha (8.0–13.0 Hz), beta 1 (13.0–22.0 Hz) and beta 2 (22.0–32.0 Hz) frequency bands. The duration of analysis epochs was 10 s. Spectral analysis was carried out using the Neuron-Spectrum.NET software (Neurosoft, Ivanovo, Russia). The average amplitude of the spectrum in a specific frequency range (alpha, beta, theta and delta rhythms) and rhythm index (the percentage of time that EEG activity is present in the EEG sample) were calculated separately in each particular range based on the power spectrum obtained on the basis of the fast Fourier transform algorithm.

### 2.6. Comet Assay

After decapitation of rats under light ether anesthesia, blood samples were taken to analyze the level of fragmentation and global DNA methylation. According to the method [21], cells were isolated from the collected material, and cell suspensions were prepared for further analysis. Fragmentation was studied using the DNA comet method [21]. Whole-genome DNA methylation was assessed using the method of DNA comets with modifications using restriction enzymes MspI and HpaII [22]. Cell suspensions (55 μL) were added to a 1% solution of low-melting agarose (500 μL) in phosphate-buffered saline (PBS) and applied to glasses precoated with 1% universal agarose and incubated with a coverslip on ice for 10 min. After the agarose solidified, the glasses were placed in a lysis buffer (10 mM TrisHCl pH 10, 2.5 M NaCl, 100 mM EDTA-Na, 1% Triton X100, 10% DMSO) and incubated for at least 1 h at 4 °C. After incubation, the glasses were washed 3 times with a solution of 10 mM EDTA with 5% DMSO in PBS for 10 min. Then, 100 μL of a solution containing 1 U HpaII or 1.5 U MspI (SibEnzyme, Novosibirsk, Russia) with reaction buffer was applied on the glasses and incubated in a humid chamber for 1 h at 37 °C. Then, alkaline electrophoresis was carried out in a solution (0.3 M NaOH and 1 mM EDTA-Na, pH13) for 20 min at a field strength of 1 V/cm, and the glasses were fixed in 70% ethanol (20 min), dried, and stored at room temperature. For the same test DNA sample, three variants were used in the experiment: one with MspI, one with HpaII, and one without the addition of enzymes. The latter option served as a control over the preservation of DNA in the reaction buffer. The preparations were stained by SYBRGreenI, and registration was carried out on OLYMPUS BX-52 (Tokyo, Japan) microscope combined with an OLYMPUS RX-420 (Tokyo, Japan) digital camera at a magnification of ×100. Images of DNA comets (100 cells from each animal) were analyzed using the CASP 1.2.2 program.

The percentage of DNA fragments in the tail of comets (“% DNA in the tail”) was used as an indicator of DNA damage. For each glass, about 100 cores were analyzed. The percentage of methylation was calculated using the following formula: 100 − (HpaII/MspI × 100), where HpaII and MspI are the average percentage of DNA in the comet’s tail in 100 nuclei on preparations treated with HpaII and MspI, respectively.

### 2.7. Statistical Analyses

Statistical analysis of the research results was carried out using the Statistica 6.1 software package. The Shapiro-Wilk W-test was used to decide on the type of feature distribution. To compare groups, we used the Mann-Whitney U-test. Null hypotheses about the absence of differences between the groups were rejected at the achieved significance level of *p* ≤ 0.05.

## 3. Results

### 3.1. Monitoring of Peat Combustion Product Concentration in Exposure Chamber

The average concentrations of combustion products in the exposure chamber were as follows: PM_2.5_—0.92 ± 0.34 mg/m^3^; CO—40.8 ± 1.9 mg/m^3^; NO_2_—0.032 ± 0.008 mg/m^3^; SO_2_—0.003 ± 0.001 mg/m^3^; formaldehyde—0.045 ± 0.008 mg/m^3^; furfural—0.21 ± 0.08 mg/m^3^; acetaldehyde—0.57 ± 0.09 mg/m^3^. Qualitative analysis identified the following classes of compounds: aromatic hydrocarbons (benzene, toluene and xylene), terpenes, terpenoids, phenol and its derivatives, aldehydes and carboxylic acids (acetic acid, pentadecanoic acid and palmitic acid).

**Figure 1:**
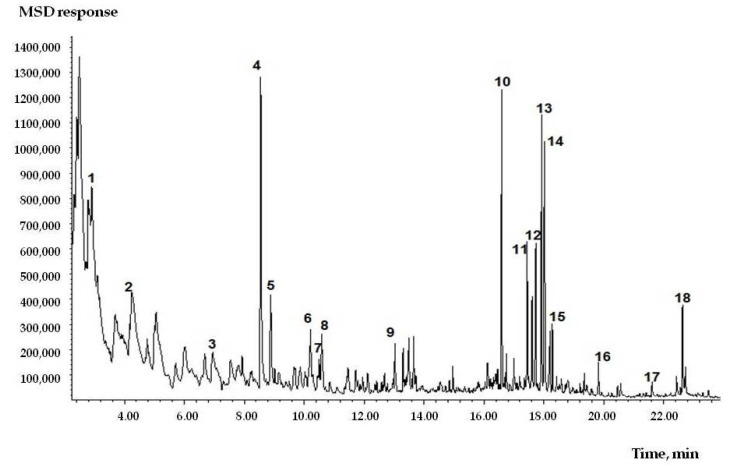
Chromatogram of peat combustion products taken from the air on SPME microfiber. Identified components: 1—benzene, 2—toluene, 3—o-xylene, 4—α-pinene, 5—camphene, 6—3-carene, 7—o-cymene, 8—D-limonene, 9—endo-borneol, 10—longifolene, 11—gamma-muurelen, 12—α-muurelen, 13—gamma-cadinene, 14—δ -cadinene, 15—α-calocoren, 16—cadalene, 17—pentadecanoic acid, 18—palmitic acid.

As can be seen in Figure 1, the air contains aromatic hydrocarbons (benzene), terpene hydrocarbons (α-pinene, longifolene, γ-cadinene and δ-cadinene), aldehydes (formaldehyde, acetaldehyde, furfural and 5-methylfurfural), and phenol derivatives (o-guaiacol).

**Figure 2:**
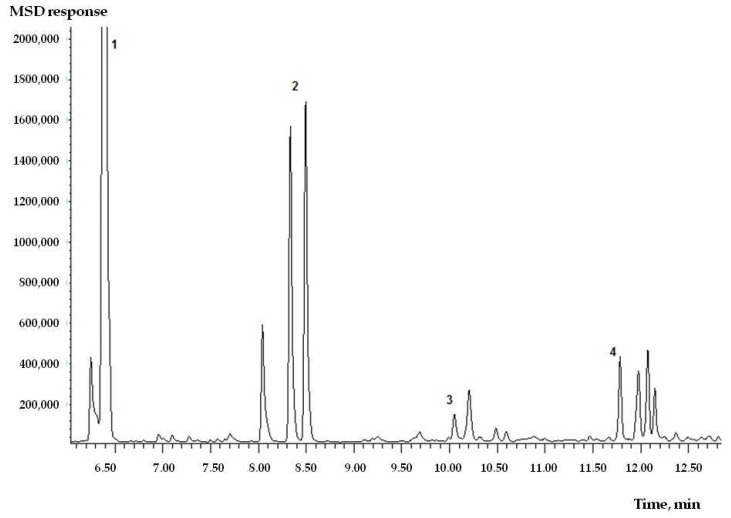
Chromatogram of derivatized aldehyde PFBHA. Identified components: 1—formaldehyde, 2—acetaldehyde, 3—propionic aldehyde, and 4—methylglyoxal.

The results of qualitative analysis for volatile aldehydes showed significant selection of formaldehyde and acetaldehyde (Figure 2).

### 3.2. Behavioral Effects of Peat Smoke

The results of the open-field test of rats exposed to peat smoke are presented in Table 1. The results show that exposure to peat combustion products for 24 h leads to a change in the behavior indicators of experimental animals in the open field. Rats exposed to peat smoke explored 55.1% of the total arena area, while control animals explored 36.4% of the arena (U = 50; Z = −2.56; *p* = 0.011; Table 1). The total distance traveled by the exposed animals during the test was calculated as 643 сm, while the control animals covered only 487 сm (U = 71; Z = −1.68; *p* = 0.092; Table 1). We observed significant increase in anxiety in exposed rats, which was manifested in an increase in the number of acts of “freezing” (U = 15; Z = −1.97; *p* = 0.048; Table 1), as compared with the control group. Together with the increased number of freezing acts, the observed increase in motor activity in the exposed animals should be considered as an indicator of high stress levels and violation of inhibitory processes.

**Figure 3:**
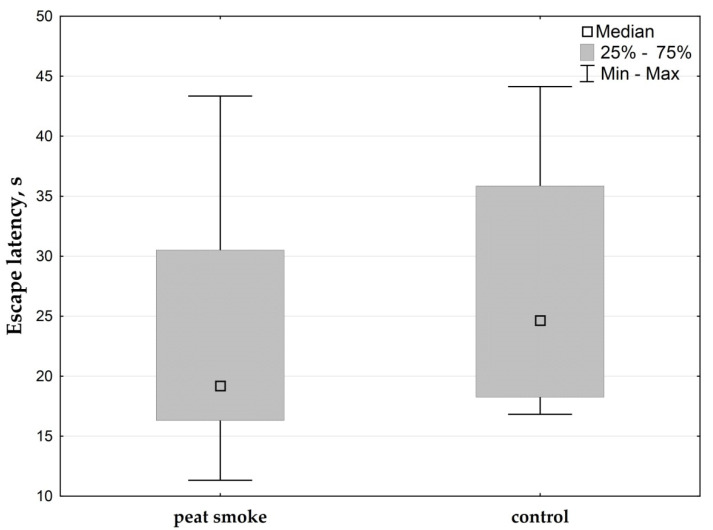
Results of Morris water maze test.

The assessment of spatial learning and memory in rats was carried out using the Morris water maze. No significant differences in escape latency were observed between groups (Figure 3).

### 3.3. EEG Analysis

EEG results showed a statistically significant increase in the index and average amplitude of the δ-rhythm in the right hemisphere in rats exposed to peat smoke in comparison with the corresponding indicators of the control group (U = 4.0; Z = 2.71; *p* = 0.006 and U = 4.0; Z = 2.72; *p* = 0.006, respectively; Table 2). A decrease in the index of the beta-2 rhythm in exposed rats was revealed in the left hemisphere compared to the control group (U = 10.0; Z = −2.22; *p* = 0.026; Table 2).

### 3.4. Assessment of DNA Fragmentation and Global DNA Methylation in Rat Blood

No statistically significant differences were found in the level of DNA fragmentation in rats exposed to peat smoke compared with the control group (Table 3). At the same time, a statistically significant decrease in the level of global DNA methylation in the blood of exposed rats was observed (*p* = 0.0001, Table 3).

## 4. Discussion

It is known that toxic gases and aerosols contained in the smoke of wildfires have a negative impact on human health [12]. At the same time, there is a lack of information about the effect of wildfire smoke on the state of the nervous system at levels and durations of exposure close to those of real smoke. The neurotoxic effects of acute CO poisoning, characterized by impairment of complex brain functions, such as perception, processing and analysis of information, memorization, and learning, which may appear sometime after cessation of exposure [23,24,25], have been widely studied. Experimental modeling of smoke from a peat fire for 24 h revealed a neurotoxic effect characterized by changes in behavior and electrophysiological indicators in rats, indicating the formation of an increased level of stress, which goes beyond physiological adaptation. The results obtained in the current study are consistent with research conducted by Amendola et al. (2019), which showed that the accumulation of CO_2_ leads to hypercapnia and respiratory acidosis, as a result of which the brain regions, ion channels, and neurotransmitters involved in the formation of negative emotional reactions in rats are activated [26].

The observed changes in the EEG parameters of control rats are consistent with numerous studies describing the effect of different anesthetics on EEG signals. EEG slowing associated with anesthesia (for example, when using isoflurane, sevoflurane, propofol, medetomidine, etc.) is associated with a decrease in EEG beta activity (13–30 Hz) and an increase in both EEG alpha activity (8–12 Hz) and delta activity (0–4 Hz) [27,28,29,30,31]. Changes in the bioelectric activity of rats exposed to peat smoke were characterized by an increase in the index and frequency of low-frequency rhythms in the delta band as well as a decrease in those in the beta band compared to the control group under similar anesthesia conditions.

Despite the fact that wildfire smoke is a multicomponent mixture of gases and particles with proven mutagenic or genotoxic properties [32,33], the level of DNA fragmentation in blood cells did not show statistically significant differences when compared with the control. Studies conducted by Ambatipudi et al. (2016) show smoking-related changes in DNA methylation in the blood [34]. These data are consistent with studies conducted by Tsaprouni (2014) and Zeilinger (2013), indicating that tobacco smoke exposure significantly affects the DNA methylation levels in whole blood, and these changes are largely corrected after smoking cessation [35,36]. DNA methylation is considered one of the important mechanisms in epigenetics, as it affects the expression of genes, “turning them on or off” [37]. It is believed that DNA methylation is a dynamic process that is influenced by environmental factors (diet, exercise, smoking and air pollution) at different periods of ontogenesis [38]. Findings from a cross-over trial of controlled human exposure to concentrated ambient particles (CAPs) have suggested that PM can determine the loss of methylation in blood DNA, potentially reflecting activation of proinflammatory conditions in blood leukocytes [39]. The data of Baccarelli show that blood leukocyte DNA methylation decreases rapidly following peaks of high ambient levels of traffic particles [40]. Previous in vitro experiments have shown that PM-induced oxidative stress [41,42] may produce genomic hypomethylation [43]. The hypomethylation DNA in blood we observed may be a consequence of alveolar inflammation and the release of inflammatory mediators upon inhalation of solid particles [44], and it may also be due to the direct effect of toxic smoke components on blood cells by inducing oxidative stress or inflammatory reactions [42,43]. Сellular heterogeneity affects the results of DNA methylation measurements using whole blood DNA [45]. It is likely that counting cells of each type (for example, neutrophils or lymphocytes) in a statistical analysis may be sufficient to correct the confounding effect of cellular heterogeneity on DNA methylation measurements. However, our task at this stage was to establish the very fact of a change in the methylation profile in blood cells. The fact of genome-wide DNA hypomethylation in peripheral blood cells established by us requires further study of the contribution of epigenetic changes to the pathogenesis of disorders of the functional state of the central nervous system when rats are exposed to the smoke of a peat fire, as well as long-term consequences on the offspring.

## 5. Conclusions

The analysis of the results of our experimental study shows that 24 h exposure to peat smoke causes behavioral impairments in rats. An increase in motor activity and anxiety, as well as a change in the bioelectric activity of the brain, was revealed, which may indicate a high stress level of animals and a violation of inhibitory processes. We observed hypomethylation of DNA in the blood cells of rats exposed to smoke, and this indicator may be a marker of the effect of smoke from peat fires. In conclusion, our results indicate that understanding the CNS effects of peat smoke is becoming an integral part of health risk assessment.

## Figures and Tables

**Table 1:** Behavior analysis of rats exposed to peat smoke in the open-field test, Me (LQ; UQ).

Behavior Parameter	Control	Peat Smoke
Distance ran, cm	487 (394; 654)	643 (517; 783)
Time in the center, %	1.2 (0.3; 5.2)	3.8 (0.9; 8.5)
% area explored	36.4 (31.6; 40.7)	55.1 (39.6; 60.6) *
Rearing	12 (9; 15)	10 (5; 17)
Freezing	0 (0; 1)	1.5 (0.5; 3) *
Defecation	0 (0; 2)	1 (0; 1.5)
Grooming	1 (0; 2)	0.5 (0; 2)

Note: Me (LQ; UQ) is median and interquartile ranges; * differences are statistically significant compared to control at *p* < 0.05.

**Table 2:** EEG examination results, Me (LQ; UQ).

	Average Amplitude, μv		Rhythm Index, %	
	Peat Smoke	Control	Peat Smoke	Control
Bands	Left-side brain			
δ	60.3 (39.8; 62.7)	38.7 (27.7; 57.8)	0.8 (0.4; 1.0) *	0.3 (0.2; 0.4)
θ	49.2 (47.9; 55.5)	50.2 (48.2; 52.0)	11.3 (9.6; 11.9)	9.90 (5.9; 11.2)
α	35.1 (34.0;4 1.5)	37.7 (34.5; 41.1)	27.9 (25.2; 32.9)	23.4 (20.4; 31.8)
β1	21.0 (19.8; 23.3)	20.4 (18.0; 22.5)	17.6 (16.9; 18.9)	18.3 (17.4; 20.2)
β2	12.3 (10.6; 16.1)	13.6 (10.8; 15.2)	15.2 (12.2; 22.5) *	27.9 (19.8; 31.9)
	Right-side brain			
δ	71.9 (57.9; 74.5) *	41.0 (19.3; 52.2)	0.9 (0.5; 1.5) *	0.2 (0.1; 0.4)
θ	49.8 (49.3; 53.2)	55.4 (50.1; 56.1)	11.1 (9.5; 11.9)	10.3 (3.7; 11.6)
α	37.2 (34.5; 37.7)	37.5 (34.9; 40.0)	27.2 (24.0; 29.4)	23.3 (19.4; 28.7)
β1	20.0 (18.9; 22.2)	21.9 (18.3; 26.4)	18.2 (16.6; 20.6)	18.9 (17.9; 2.30)
β2	12.5 (11.9; 14.7)	13.8 (11.7; 14.5)	17.9 (17.8; 23.0)	29.2 (20.7; 36.7)

Note: * differences are statistically significant compared to control at *p* < 0.05.

**Table 3:** DNA fragmentation and global DNA methylation in blood of rats.

Indicators	Control	Peat Smoke
DNA fragmentation, %	4.68 (3.4; 5.97)	3.58 (0.37; 8.31)
Global DNA methylation, %	78.18 (51.6; 97.76)	46.94 (13.87; 64.41) *

Note: Me (LQ; UQ) is median and inter-quartile ranges; * differences are statistically significant compared to control at *p* < 0.05.

## Data Availability

The data presented in this study are available on request from the corresponding author.
